# Exploring Metabolic and Immunological Biomarkers for Oral Squamous Cell Carcinoma: Potential Targets for Precision Therapy

**DOI:** 10.3390/biology14091109

**Published:** 2025-08-22

**Authors:** Rajneesh, Rahul Tiwari, Vishal Kumar Singh, Awnish Kumar, Sanjana Mehrotra, Vibhav Gautam, J. F. Neville, Vyomika Bansal, Rajiv Pathak, Akhilesh Kumar Singh, Rajiv Kumar

**Affiliations:** 1 Centre of Experimental Medicine & Surgery, Institute of Medical Sciences, Banaras Hindu University, Varanasi 221005, India; rajneeshbhu15@gmail.com (R.); rahul.tiwari@bhu.ac.in (R.T.); vishalsingh.gkp009@gmail.com (V.K.S.); kumarawnish786@gmail.com (A.K.); vibhav.gautam4@bhu.ac.in (V.G.); 2 Department of Human Genetics, Guru Nanak Dev University, Amritsar 143005, India; sanjana.human@gndu.ac.in; 3 Department of Surgical Oncology, Institute of Medical Sciences, Banaras Hindu University, Varanasi 221005, India; drnevillejf@bhu.ac.in; 4 Oral & Maxillofacial Surgery Unit, Faculty of Dental Sciences, Institute of Medical Sciences, Banaras Hindu University, Varanasi 221005, India; bansalvyomika@gmail.com; 5 Department of Genetics, Albert Einstein College of Medicine, Bronx, New York, NY 10461, USA; rajiv.pathak@einsteinmed.edu

**Keywords:** oral squamous cell carcinoma, metabolome, proteome, biomarkers, therapeutic targets, immunotherapy

## Abstract

Oral squamous cell carcinoma (OSCC) is a malignant neoplasm of the oral epithelium that constitutes majority of oral cancers and is strongly associated with risk factors such as tobacco use and genetic alterations. Mortality rates for OSCC are high because many cases are misdiagnosed or identified at later stages, and treatment options are limited with high recurrence. Therefore, there is an urgent need for improved diagnostic and therapeutic strategies. OSCC tumor cells, like those in other solid malignancies, exhibit significant alterations in metabolic pathways and the immune microenvironment. These changes can serve as valuable biomarkers for early detection and as targets for innovative treatment strategies. This review summarizes the current understanding of metabolic and immunological biomarkers that are either currently in use for OSCC or are in different phases of clinical trials.

## Simple Summary

Oral squamous cell carcinoma (OSCC) is a major global health challenge due to late diagnosis and limited treatment options, resulting in high mortality. This review explores how changes in the metabolic and immune microenvironment of OSCC can serve as biomarkers for early detection and as targets for precision therapies. By providing metabolomic and immunological insights, we aim to guide the development of non-invasive diagnostics and personalized treatments to improve patient outcomes.

## 1. Introduction

Oral squamous cell carcinoma (OSCC) originates in the squamous epithelium of the oral cavity and is an aggressive malignancy with a high propensity for regional and distant metastasis via the lymphatic system [1,2]. According to GLOBOCAN 2020 data, and corroborated by multiple sources, the global incidence of OSCC was approximately 370,000 new cases and 177,000 deaths [3]. It is particularly prevalent in the Asian populations, and key risk factors include smoking, alcohol consumption, smokeless tobacco use, and betel quid chewing [4,5,6,7,8,9,10]. Genetic susceptibility, viral infections, and chronic inflammatory conditions also contribute to OSCC etiology [4,11,12,13].

Survival rates for patients with OSCC vary widely depending on the stage at diagnosis. Early-stage (stage I) detection is associated with high survival rates (about 80%), but these rates drop to 20–30% for late stage (stage III–IV) diagnosis [14]. Due to asymptomatic progression or nonspecific early symptoms, most OSCC cases are diagnosed at an advanced stage, resulting in poor prognosis and high treatment costs [15,16]. While histopathology remains the gold standard for the diagnosis, biomarkers based on DNA, RNA, metabolites, proteins, immune-related molecules, and microbial compounds have shown great potential for early detection and prognosis, though they are not yet part of routine clinical practice [17].

This narrative review provides an up-to-date, in-depth summary of important metabolic and immunological biomarkers in OSCC, emphasizing how alterations in glycolysis, amino acid metabolism, and lipid metabolism alongside immune interactions shape disease progression and therapeutic response. The literature search was conducted using broad queries across key scientific databases such as PubMed, Scopus, and Google Scholar, focusing on recent publications up to 2024. Search terms included combinations of “oral squamous cell carcinoma,” “OSCC”, “metabolic biomarkers,” “immunological biomarkers,” “metabolomics,” and “immunotherapy.” The literature was selected based on relevance, recency, and an emphasis on translational and clinical significance.

Unlike previous reviews, we offer a comprehensive integration of these biomarker classes, with particular focus on their crosstalk, translational challenges, and the promise of non-invasive diagnostics, multi-omics, and AI-driven approaches for advancing clinical practice. By systematically examining how metabolic reprogramming and immune microenvironmental changes converge in OSCC, we aim to bridge the gap between biomarker discovery and clinical implementation, guiding the development of more effective strategies for early detection and personalized therapy.

## 2. Metabolic Alterations and Therapeutic Targeting in OSCC

Metabolic reprogramming is a central hallmark of OSCC, driving tumor cell survival, proliferation, and metastasis [18]. These metabolic alterations generate a distinct molecular profile, often detectable before clinical symptoms arise, thus presenting valuable opportunities for early diagnosis and therapeutic intervention [19]. Metabolomics—the comprehensive analysis of small molecules and metabolites in cells, tissues, or biofluids—offer critical insights into the altered physiology of cancer patients [20]. Technological advances in metabolomic profiling, including nuclear magnetic resonance (NMR) spectroscopy, mass spectrometry (MS), tandem MS, gas chromatography (GC), and high-performance liquid chromatography (HPLC) now enable the detection of the metabolite at extremely low concentrations with high sensitivity and specificity [21,22].

**Figure 1:**
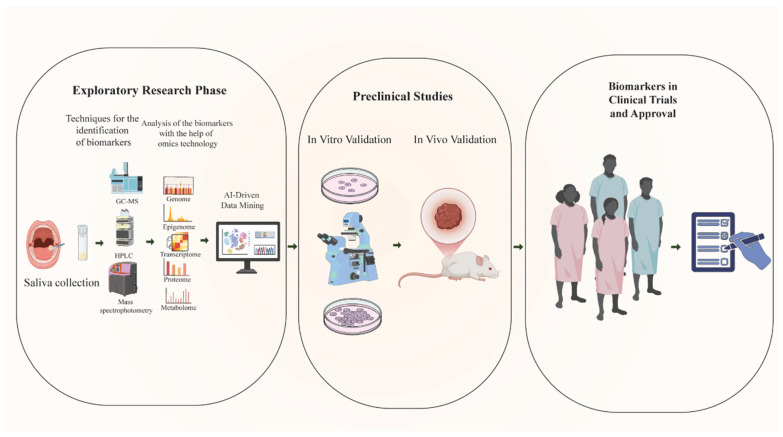
Schematic representation of the biomarker validation pipeline for OSCC. The workflow begins with the exploratory research phase, where saliva samples are collected and analyzed using advanced analytical techniques such as GC-MS, HPLC, and mass spectrophotometry. Identified candidate biomarkers undergo comprehensive characterization through multi-omics approaches, including genomics, epigenomics, transcriptomics, proteomics, and metabolomics, with AI-driven data mining used to prioritize promising markers. In the preclinical studies phase, biomarkers are validated first in vitro using cell culture and laboratory assays, followed by in vivo validation in animal models. The final phase involves clinical trials and regulatory approval, where biomarkers are evaluated in human cohorts for their diagnostic or prognostic utility before potential implementation in clinical practice. [Abbreviations: GC-MS, gas chromatography mass spectrometry; HPLC, high-performance liquid chromatography].

A key gap in OSCC diagnostics is the inability of current methods to reliably distinguish between potentially precancerous oral lesions, such as Oral Erosive Lichen Planus (OELP), Oral Lichen Planus (OLP), and Oral Leukoplakia (OLK). While OELP and OLP are autoimmune inflammatory conditions, OLK is characterized by white patches with a higher risk of malignant transformation to OSCC [23]. Existing diagnostic approaches rely heavily on invasive biopsies and subjective histopathology evaluation, which are insufficient for early and accurate detection. Salivary and plasma biomarkers reflecting altered amino acid, polyamine, and choline metabolism have shown significant promise for OSCC detection (Table 1). Notably, metabolites associated with dysfunctional choline metabolism are implicated in promoting tumor malignancy [24]. Studying these metabolic irregularities not only facilitate early detection but also provides insights into the disease mechanism and support the development of targeted therapies (Figure 1).

### 2.1. Dysfunctional Amino Acid Metabolism

Altered amino acid (AA) metabolism is a defining feature of OSCC, impacting both disease progression and diagnostic strategies [25]. Plasma free amino acid (PFAA) profiling using HPLC reveals that OSCC patients have reduced levels of aspartate, glutamate, and arginine, alongside elevated asparagine, glutamine, and cysteine. These metabolic shifts vary with tumor differentiation; for example, metabolites such as serine and asparagine distinguish well-differentiated from moderately differentiated tumors [26]. Mendelian randomization studies further identify tryptophan and pentadecanoate as metabolites linked to increased OSCC risk, while uridine and alpha-tocopherol show protective effects [27]. Non-invasive diagnostic approaches leverage these metabolic changes. Ultra-high-performance liquid chromatography coupled with quadrupole-orbitrap high-resolution mass spectrometry (UHPLC/Q-Orbitrap HRMS) has identified a plasma biomarker panel (decanoylcarnitine, cysteine, and cholic acid) that distinguishes OSCC from OELP with near-perfect accuracy (AUC = 0.998) [28]. Similarly, urine metabolite analysis highlights 6-hydroxynicotinic acid and valine as markers for distinguishing OSCC from healthy controls with high sensitivity (94.4%) and specificity (91.4%). A combination of 6-hydroxynicotinic acid, cysteine, and tyrosine achieves 92.7% accuracy in distinguishing OSCC from OLK [29].

Glutamine metabolism is particularly pivotal in OSCC, fueling nucleotide synthesis (purines and pyrimidines), glycosylation via glucosamine-6-phosphate (GlcN6P), and redox homeostasis through glutamate conversion which enters the TCA cycle, generates NADPH (via glutamate dehydrogenase) to mitigate oxidative stress, and supports glutathione biosynthesis, a critical antioxidant defense [30,31]. OSCC cells often overexpress the glutamine transporter SLC1A5/ASCT2, enhancing glutamine uptake. Inhibiting this transporter depletes intracellular glutamine, impairs tumor proliferation, and highlights a promising therapeutic target [32,33]. Additionally, tryptophan metabolism via the kynurenine pathway plays a significant role in OSCC immune evasion by enhancing regulatory T cell (Treg) function. This metabolic reprogramming contributes to tumor immune suppression and represents another avenue for therapeutic intervention [34].

### 2.2. Lipid-Choline Metabolism

Choline metabolism is a crucial component of lipid metabolism, and its disruption is strongly linked to the development of OSCC. In OSCC, choline metabolism becomes activated, leading to elevated levels of phosphatidylcholine and phosphatidylethanolamine—a key member for membrane synthesis and tumor growth. Metabolomic profiling using NMR and UHPLC/Q-Orbitrap HRMS has revealed significant alterations in choline-related pathways, notably reduced free choline and increased trimethylamine N-oxide in serum, both of which serve as metabolic signatures of OSCC. The diagnostic potential of choline derivatives has been highlighted by studies demonstrating that metabolomic analysis of saliva and serum can effectively detect these disruptions during oral carcinogenesis [35]. Furthermore, research has consistently shown a strong association between altered choline metabolism and OSCC, as evidenced by changes in salivary choline levels in affected patients [36,37]. Salivary metabolomics identifies propionylcholine as a biomarker for early-stage OSCC (sensitivity of 76.9% and specificity of 96.7%, *p* < 0.0001), while phosphorylcholine correlates with advanced disease. These choline derivatives, detectable in non-invasive saliva and plasma samples, enable differentiation of OSCC from healthy controls and precancerous lesions (e.g., OLK) with high accuracy (AUC = 0.997 for multi-marker panels) [38,39]. While these findings are promising, further validation in larger and more diverse patient cohorts is warranted before clinical implementation.

Beyond choline metabolism, lipid metabolism, as a whole, is significantly dysregulated in OSCC. This is driven by the rapid proliferation of cancer cells, which requires increased lipids for membrane synthesis, energy production, and signaling [40]. This heightened demand depletes cellular lipid reserves, fueling tumor initiation, progression, and resistance to therapy [18]. Altered pathways involving lipid uptake, biosynthesis, transport, and degradation enhance OSCC aggressiveness. Fatty acid (FA) uptake, mediated by CD36 and FA-binding proteins (FABPs), promotes OSCC proliferation and metastasis. Inhibiting CD36, a key FA translocase, limits FA uptake, reducing tumor migration and lymph node metastasis [41,42]. Similarly, FABP5 enhances invasion by upregulating matrix metalloproteinase-9 (MMP-9) [43].

**Figure 2:**
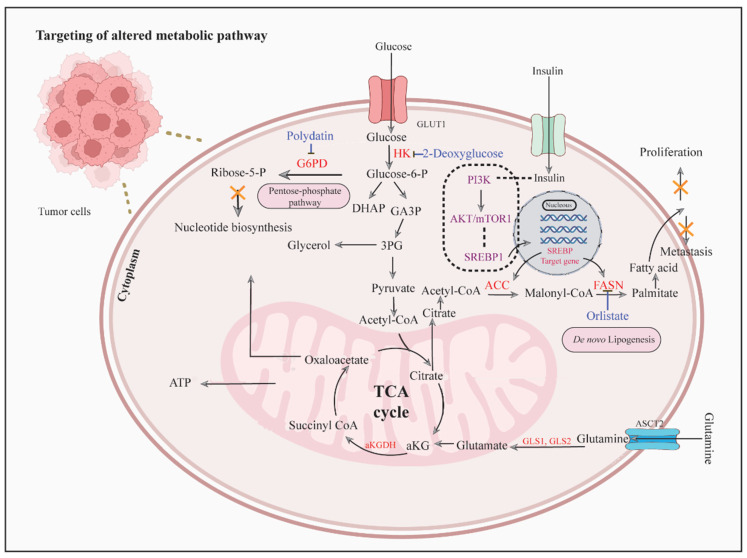
Overview of altered metabolic pathways in OSCC and their therapeutic targeting. The figure illustrates key metabolic processes supporting OSCC cell growth and survival, including glycolysis, PPP, the TCA cycle, and de novo lipogenesis. Glucose uptake via GLUT1 fuels glycolysis, with HK-II catalyzing the first step; inhibition by 2-deoxyglucose disrupts this pathway. The PPP, regulated by G6PD, generates ribose-5-phosphate for nucleotide biosynthesis and is targeted by polydatin. The PI3K/AKT/mTOR/SREBP1 signaling axis promotes de novo lipogenesis through upregulation of ACC and FASN, FASN can be inhibited by an agent such as orlistat, thereby reducing fatty acid synthesis, proliferation, and metastasis. Glutamine metabolism, mediated by ASCT2 and glutaminases (GLS1/GLS2), feeds into the TCA cycle to support energy production and biosynthesis. Pharmacological inhibition at these metabolic nodes is highlighted as a strategy to suppress tumor growth and metastatic progression in OSCC. [Abbreviations: HK-II, hexokinase; 2-DG, 2-deoxyglucose; GLUTs, glucose transporters; G6PD, glucose-6-phosphate dehydrogenase; PPP, pentose phosphate pathway; 3PG, 3-phosphoglycerate; GA3P, glyceraldehyde-3-phosphate; DHAP, dihydroxyacetone phosphate; aKG, alpha ketoglutarate; TCA, tricarboxylic acid cycle; α-KGDH, alpha-ketoglutarate dehydrogenase; ACC, acetyl-CoA carboxylase; FASN, fatty acid synthase; GLS1, glutaminase 1 and GLS2, glutaminase 2].

De novo FA synthesis, a hallmark of OSCC, supports membrane formation and lipid raft assembly. Acetyl-CoA, derived from glucose or glutamine, is converted to malonyl-CoA by acetyl-CoA carboxylases (ACC) and then to palmitate by fatty acid synthase (FASN). FASN, activated by PI3K/AKT and SREBP1 signaling, is overexpressed in advanced OSCC, correlating with poor prognosis and chemoresistance [44]. Inhibiting FASN with orlistat suppresses OSCC proliferation and metastasis (Figure 2) and increases sensitivity to cisplatin and paclitaxel by downregulating cell cycle regulators [45,46]. Beyond FASN, targeting lipid-driven signaling pathways and biogenesis-related enzymes offers additional therapeutic potential. For example, Sphingosine-1-phosphate (S1P), produced by sphingosine kinase, activates S1P receptor 2 (S1PR2) to promote OSCC migration [47]. Inhibiting S1PR2 reduces invasion, highlighting its anti-metastatic potential [48]. Targeting biogenesis regulators like SREBP1 or ACC could further disrupt lipid supply, enhancing OSCC sensitivity to therapies.

Importantly, lipid metabolism dysregulation is also implicated in the progression from OLP to OSCC, underscoring the relevance of these pathways for early intervention and prevention. Collectively, these findings underscore lipid metabolism and biogenesis as critical targets for inhibiting OSCC growth and overcoming therapeutic resistance.

### 2.3. Glycolysis and the Warburg Effect

OSCC exhibits metabolic reprogramming characterized by increased lactate production and the Warburg effect—a shift from oxidative phosphorylation to aerobic glycolysis, even in the presence of ample oxygen [49]. First described by Otto Warburg, this adaptation enhances glucose fermentation, resulting in elevated lactate production [50,51]. The accumulation of lactate not only supports rapid ATP generation for cancer cell proliferation but also acidifies the microenvironment, promoting tumor invasion and facilitating immune evasion. Lactic acid is unstable and rapidly converts back to pyruvic acid, causing an imbalance between production and clearance. This leads to pyruvate accumulation in the oral cavity or bloodstream. Clinical studies have shown that serum pyruvic acid levels are significantly higher in OSCC patients compared to healthy individuals (2.65 mg% ± 0.80 vs. 0.95 mg% ± 0.18, *p* < 0.0001), with levels increasing as the disease progresses. This highlights their potential as cost-effective biomarkers for early detection, disease severity assessment, and monitoring [52].

OSCC’s reliance on glycolysis is further evidenced by the overexpression of key glycolytic enzymes such as hexokinase II (HK-II), which catalyzes the phosphorylation of glucose to glucose-6-phosphate—a crucial regulatory step in glycolysis. HK-II is upregulated in cancer cells and can be selectively inhibited by 2-deoxyglucose (2-DG), a glucose analog that, once phosphorylated, accumulates as 2-deoxyglucose-6-phosphate and inhibits HK-II activity. This leads to reduced intracellular glucose, ATP depletion, and ultimately, cancer cell death [53,54,55,56].

In addition to glycolytic enzymes, glucose transporters (GLUTs), particularly GLUT-1 and GLUT-3, are overexpressed in OSCC, facilitating increased glucose uptake. Inhibiting these transporters, either pharmacologically or genetically, induces apoptosis and suppresses tumor growth [57,58]. Similarly, monocarboxylate transporters (MCTs), especially MCT-4, are essential for exporting lactate, maintaining intracellular pH, and supporting metabolic interactions within the tumor. Elevated MCT-4 expression is associated with increased proliferation, migration, and invasiveness in OSCC [59]. Inhibiting MCTs disrupts lactate transport, causes intracellular acidification, and reduces tumor viability.

### 2.4. Pentose Phosphate Pathway

The pentose phosphate pathway (PPP) is a critical metabolic node in OSCC, fueling tumor progression through NADPH production (maintaining redox balance) and ribose-5-phosphate synthesis (supporting nucleotide biosynthesis). Glucose-6-phosphate dehydrogenase (G6PD), the rate-limiting enzyme of the PPP, is overexpressed in OSCC and correlates with advanced disease, lymph node metastasis, and poor prognosis [60]. Mechanistically, G6PD inhibition disrupts NADPH synthesis, elevating reactive oxygen species (ROS) and inducing endoplasmic reticulum (ER) stress, which activates the IRE1α/PERK pathways, upregulates pro-apoptotic CHOP, and triggers caspase-mediated apoptosis [61,62].

Preclinical studies highlight polydatin, a natural G6PD inhibitor, as a therapeutic candidate. Polydatin suppresses metastasis by restoring E-cadherin expression, reversing epithelial–mesenchymal transition (EMT), and reducing OSCC cell migration/invasion by 60–70% [63]. In vivo, polydatin synergizes with cisplatin, amplifying ROS-mediated DNA damage and reducing tumor growth by 60% and lymph node metastasis by 80% [63]. These findings position PPP inhibition as a dual-strategy approach—disrupting metabolic adaptability while restoring chemosensitivity-making G6PD, a promising target for precision oncology in OSCC [60].

### 2.5. Others Salivary Biomarkers: Insights and Applications

Salivary metabolomics has emerged as a transformative approach for OSCC detection, offering non-invasive advantages over traditional tissue biopsies. Notably, elevated concentrations of metabolites such as glutamate, histidine, sialic acid and trimethylamine N-oxide (TMAO), malic acid, maltose, methionine, inosine hypoxanthine, guanine, guanosine, spermidine, and pipercolate have been consistently detected in the saliva of OSCC patients [37,64,65,66,67]. Among these, TMAO and methionine are recurrently elevated, underscoring their potential. Conversely, reduced urea levels, particularly observed in Japanese OSCC patients, suggest dysregulation of the urea cycle as a potential biomarker [36]. Advanced platforms like capillary electrophoresis–mass spectrometry (CE-MS) and conductive polymer spray ionization mass spectrometry (CPSI-MS) coupled with machine learning have identified diagnostic panels (e.g., choline, branched-chain amino acids, 2-oxoisovaleric acid) with accuracies up to 86.7% for distinguishing OSCC from healthy controls [38]. Multi-marker panels, such as propionylcholine, N-acetyl-L-phenylalanine, and sphinganine achieve exceptional diagnostic performance (AUC = 0.997, sensitivity = 100%, specificity = 96.7%) in distinguishing early-stage OSCC from controls [39]. Clinically, saliva-based testing reduces costs and procedural risks while enabling large-scale screening, outperforming tissue biopsies that face invasiveness, sampling bias, and complications like infection [28,68]. Tissue-based methods remain the gold standard for confirming diagnoses and evaluating targets like PD-L1 (the only FDA-approved immunotherapy biomarker). Emerging salivary biomarkers, such as decanoylcarnitine—a fatty acid oxidation intermediate—show robust diagnostic potential as part of a metabolomic panel (AUC = 0.998) in preclinical and early clinical studies, though multicenter validation is pending [38,69]. Integrating both approaches could optimize OSCC management: saliva’s accessibility enables population-level screening, while tissue analysis provides molecular precision for targeted therapy. This dual strategy holds promise for improving early detection and reducing mortality through timely intervention [28].

## 3. Immunological Biomarkers Associated with OSCC

The immune system plays a pivotal role in detecting and eliminating abnormal cancerous cells [70], involving both innate (e.g., macrophages, NK cells, dendritic cells) and adaptive (B and T lymphocytes) components [71]. However, cancer cells frequently develop mechanisms to evade immune detection, such as reducing antigen expression or creating an immunosuppressive microenvironment [72]. Immunological biomarkers provide valuable insights into tumor immune system interaction and can be detected through blood tests, imaging, and tissue analysis. These biomarkers include immune cell populations, surface protein molecules, cytokines, chemokines, antibodies, and tumor neoantigens, aiding in cancer diagnosis, prognosis, and treatment guidance [73].

Predictive biomarkers can further refine cancer treatment by classifying patients according to their likely response to therapies including chemotherapy, endocrine therapy, radiation, targeted approaches, and immunotherapy. They also help identify individuals at risk for significant toxicity, informing dose adjustments or alternative treatments [74].

Immunotherapy, particularly immune checkpoint inhibitors (ICIs), has revolutionized cancer care [75], but many patients do not respond to it or experience adverse effects. This highlights the need for reliable biomarkers to predict which tumors will respond to immunotherapy. The FDA has approved biomarkers such as PD-L1 expression, microsatellite instability, and tumor mutational burden; ongoing research aims to refine and optimize these for improved treatment precision.

### 3.1. Key Immune Biomarkers Identified in OSCC

#### 3.1.1. Cytokines

Cytokines, small proteins produced by immune and stromal cells, regulate cell growth, survival, migration, and mediate many functions. Elevated levels of specific pro-inflammatory cytokines, such as IL-1, IL-6, IL-8, and TNF-α contribute to tumor progression and invasion by promoting cell proliferation, epithelial–mesenchymal transition (EMT), and vascularization, while also enhancing the tumor’s ability to evade immune detection [76,77,78]. Conversely, anti-inflammatory cytokines, including IL-4, IL-10, and IL-13, function as immune suppressants, facilitating cancer cells’ evasion of immune surveillance [79,80,81,82]. These cytokines have been extensively studied for their diagnostic roles in OSCC. Significantly higher concentrations of TNF-α, IL-6, and IL-8 has been reported in OSCC patients, making them reliable for early detection [78,83]. However, their levels may be influenced by local inflammation and other malignant states. Certain cytokines also have prognostic value, with elevated levels indicating poor prognosis, aggressive disease, or higher likelihood of metastasis. For instance, TNF-α is associated with higher rates of metastasis and facilitates angiogenesis in head and neck squamous cell carcinoma (HNSCC) patients by enhancing the expression of vascular growth factors and increasing the activation of the ERK3 pathway [84,85]. Profiling cytokine levels can thus provide insights into patient responses to specific treatments, including immunotherapies [86]. Monitoring changes over time can assist in tracking cancer progression or treatment effectiveness.

#### 3.1.2. Chemokines

Chemokines are signaling peptides that recruit inflammatory cells and play dual roles in tumor growth and angiogenesis during inflammation [87,88]. In OSCC, the CXCL12/CXCR4 axis is involved in metastatic migration, often correlating with poor prognosis [89]. The CCR7/CCL21 pair is linked to lymph node metastasis and advanced clinical stage patients [90]. Other chemokines, such as CXCL13 and CX3CL1, have emerged as prognostic markers, with high levels associated with bone invasion and poorer outcomes [91,92,93,94]. The CCL28/CCR10 axis is also linked to tumor differentiation and aggressiveness, suggesting its potential as a prognostic biomarker [95].

#### 3.1.3. Tumor Microenvironment (TME) Components

**Figure 3:**
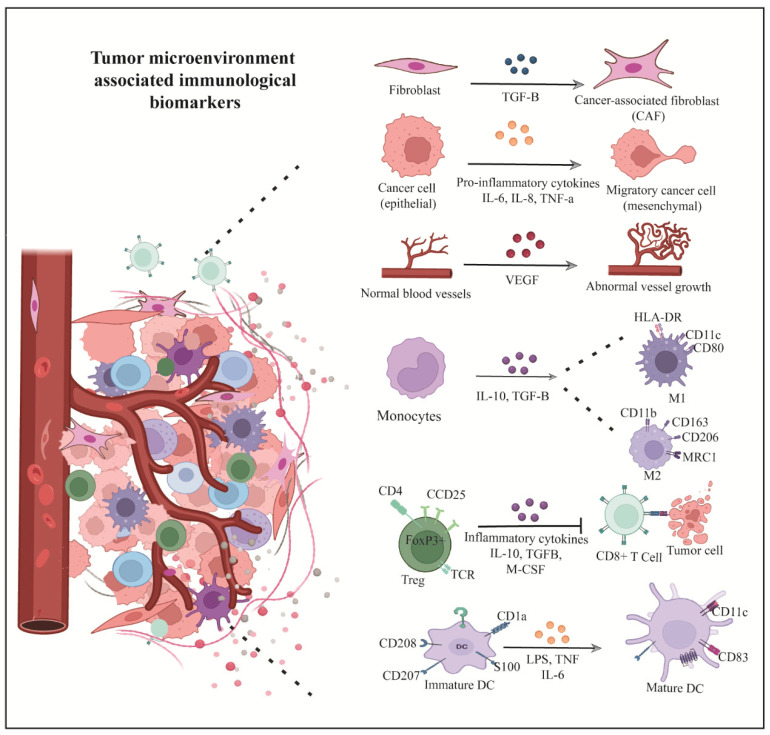
Tumor microenvironment-associated biomarkers. The intricate interplay between immune cells and the tumor microenvironment leads to the expression of various immunological biomarkers. These immune cells can be targeted for immunotherapy and can serve as biomarkers for cancer diagnosis. In OSCC, specific immune cell biomarkers, such as CD11c, CD80, HLA-DR, CD163, CD11b, CD206, and others, are used for diagnosis. Additionally, markers like CD57, CD3, CD8, and CD19^+^ B cells have prognostic potential in OSCC. Other cells, such as CAFs and endothelial cells, also express specific markers. [Abbreviations: CAFs, cancer-associated fibroblasts; TCR, T cell receptor; DC, dendritic cell].

The TME is a complex milieu of cancer, stromal, and immune cells, along with signaling molecules. It supports tumor growth and immune evasion, making it a critical focus for understanding OSCC progression and resistance [96]. Key TME components include tumor-infiltrating lymphocytes (TILs), regulatory T cells (Tregs), cancer-associated fibroblasts (CAFs) myeloid-derived suppressor cells (MDSCs), tumor-associated granulocytes, tumor-associated macrophages (TAMs), and dendritic cells (DCs). These cells can both promote tumor progression and serve as immunological biomarkers for diagnosis, prognosis, and therapy. (Table 2) (Figure 3).

The extracellular matrix (ECM) within the TME undergoes remodeling, driven by matrix metalloproteinases (MMPs), which regulate cytokine levels and mediate cell migration during inflammation and metastasis [97]. MMPs such as MMP-1, -2, -3, -7, and -9 have been extensively studied in OSCC, with MMP-9 identified as a predictive biomarker for lymph node metastasis [98]. Additionally, non-invasive detection methods, such as saliva-based MMP-1 analysis, are being explored for OSCC diagnostics, indicating their potential for clinical applications [99]. These diverse components interact dynamically to shape the immune landscape and influence tumor progression. Among these, the immune cell populations within the TME play particularly pivotal roles in modulating both anti-tumor responses and immune evasion mechanisms in OSCC.

#### 3.1.4. Tumor-Infiltrating Immune Cells

The TME of OSCC is shaped by diverse immune cell populations that influence tumor progression and therapeutic outcomes. TILs, including CD3^+^ T cells, CD4^+^ helper T cells and CD8^+^ cytotoxic T cells, play dual roles in anti-tumor immunity and immune evasion [100,101].

CD4^+^ T cells differentiate into subsets such as Th1, Th2, Th17, and T regulatory (Treg) cells, each of which modulates OSCC progression through cytokines secretion. Th1 cells, activated by IFN-α/β and IL-12, promote tumor suppression via IFN-γ and TNF-α [102,103]. In contrast, Th2 cells drive pro-tumor M2 macrophage polarization through IL-4 [104]. Tregs, identified by FOXP3 and CD25 expression, suppress cytotoxic T cell activity, correlating with immune evasion and poorer prognosis [105].

CD8^+^ T cells, though critical for tumor cell lysis via IFN-γ and granzyme secretion, are often hindered by checkpoint molecules like CTLA-4 and PD-1, contributing to variable survival outcomes [106,107]. CAFs marked by α-smooth muscle actin (αSMA) and fibroblast activation protein (FAP) remodel the ECM, secrete pro-angiogenic factors, and recruit immunosuppressive cells, linking their overexpression to therapy resistance and reduced survival [108,109,110,111,112].

MDSC, characterized by markers CD11b and CD33, further dampen anti-tumor immunity by inhibiting T cell activation and promoting EMT. Elevated MDSC infiltration correlates with malignant progression and poor prognosis. Tumor-associated macrophages (TAMs) exhibit phenotypic plasticity within the TME [113].

TAMs exhibit phenotypic plasticity within the TME. Initially, bone marrow-derived monocytes adopt an M1 phenotype, secreting IL-6, IL-12, and TNF to enhance CD8^+^ T/NK cells [114,115,116]. However, hypoxia and CSF-1 drive a shift toward immunosuppressive M2 TAMs, marked by CD163, CD206, and MRC1, which promote metastasis and correlate with advanced disease [117,118,119,120,121].

In addition to immune cell composition, the physical and chemical conditions of the TME—particularly hypoxia—further modulate tumor behavior and immune responses in OSCC.

**Table 2:** Lists key TME cell markers and their prognostic roles.

Cell Type	Markers	Role	Clinical Significance	Reference
CD8^+^ T cells	CD8	Anti-tumor	Good prognosis	Mittrücker et al., 2014 [106]
Tregs	FOXP3, CD25	Immunosuppression	Poor prognosis	Chaudhary & Elkord, 2016 [105]
M2 TAMs	CD163, CD206	Tumor promotion	Poor prognosis	Chaudhari et al., 2020 [121]
CAFs	αSMA, FAP	ECM remodeling, immunosuppression	Poor prognosis	Dourado et al., 2018 [111]
MDSCs	CD11b, CD33	Immune suppression	Disease progression	Pang et al., 2020 [113]

#### 3.1.5. Hypoxia and Inflammation Mediators as Biomarkers

Hypoxia and inflammation are pivotal features of the TME tumor microenvironment in OSCC, profoundly influencing disease progression and biomarker development. Hypoxic conditions within the tumor, mediated by HIFs, especially HIF-1α, drive the expression of genes involved in metabolism, angiogenesis, ECM remodeling, and immune modulation, and are strongly associated with poor survival, lymph node metastasis, and adverse prognosis in OSCC patients [122,123,124,125,126,127,128]. In parallel, inflammatory mediators such as cytokines and acute-phase proteins orchestrate a dual role by activating anti-tumor immune responses while also promoting tumor growth and immune evasion. Systemic inflammatory markers—including neutrophil–lymphocyte ratio (NLR), lymphocyte–monocyte ratio (LMR), platelet–lymphocyte ratio (PLR), and C-reactive protein (CRP)—have emerged as accessible prognostic biomarkers, with elevated NLR and CRP correlating with advanced disease and poor outcomes, and altered LMR and PLR reflecting immune status and tumor aggressiveness [97,129,130]. Molecular mediators such as NF-κB and cyclooxygenase-2 (COX-2) further modulate the inflammatory milieu, facilitating angiogenesis, tumor proliferation, and resistance to apoptosis, while also serving as potential markers of disease progression and therapeutic targets [131,132,133]. Together, hypoxia-driven pathways and inflammatory mediators not only shape the OSCC microenvironment but also provide a robust foundation for developing integrated biomarker panels to improve diagnosis, prognostication, and personalized therapy.

## 4. Altered Immunological Biomarkers as Therapeutic Target

Traditional treatments for OSCC—including surgery, radiotherapy, and chemotherapy—are effective for early-stage disease but are often limited by invasiveness, resistance, and adverse effects. The advent of immunotherapy has transformed the therapeutic landscape by harnessing the immune system to recognize and eliminate cancer cells. Strategies such as immune checkpoint inhibitors (ICIs), adoptive cell therapy (ACT), antibody-based treatments, cytokine therapy, tumor vaccines, and gene therapy aim to stimulate or modulate anti-tumor immunity and enhance immune cell infiltration into the tumor microenvironment [134]. Among these, ICIs targeting PD-1, PD-L1, and CTLA-4 have shown promising response rates and improved survival, particularly in recurrent or metastatic OSCC [135]. However, not all patients respond to these therapies, highlighting the need for reliable biomarkers to predict therapeutic response and guide personalized treatment.

### 4.1. Immune Check Points Inhibitors

**Figure 4:**
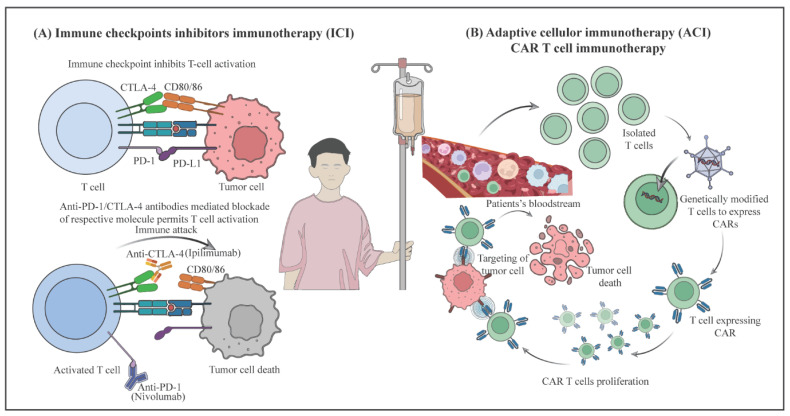
Innovative immunotherapeutic approaches for OSCC. The TME induces the expression of ICM, including PD-1 and CTLA-4 inhibitors, which hamper T cell activation and effector functions. These ICM can be blocked using monoclonal antibodies to prevent their ligation with the corresponding ligands, such as CD80/86 and PD-1. Inhibiting ICM enhances anti-tumor immunity (**A**). CAR T cell therapy: Patient T cells are engineered to express CAR targeting tumor antigens, then reinfused to improve tumor recognition and effector function (**B**). [Abbreviations: TME, tumor microenvironment; ICM, immune checkpoint molecules; PD-1, programmed cell death protein 1; CTLA-4, cytotoxic T-lymphocyte-associated protein 4; CAR, chimeric antigen receptors].

Immune checkpoints (ICs) are regulatory molecules that maintain immune activation and self-tolerance, preventing autoimmunity, and protecting host tissues from immune–mediated damage [136]. Tumors exploit these pathways to evade immune surveillance. In recent years, ICIs have become pivotal therapeutic agents for various cancers, including OSCC, by targeting checkpoint molecules such as PD-1, PD-L1, and CTLA-4, highlighting the significant role of immunosuppression in OSCC pathology [135] (Figure 4).

CTLA-4 is a transmembrane receptor primarily expressed on T cells and Tregs, where it plays a crucial role in inhibiting costimulatory signals and dampens T cell responses [137,138]. By binding to the B7 protein (CD80/CD86) on antigen presenting cells, CTLA-4 outcompetes the stimulatory receptor CD28, thereby inhibiting the costimulatory signals required for full T cell activation and leading to T cell dysfunction [138,139]. This immunosuppressive mechanism is essential for maintaining immune homeostasis but can be exploited by tumors to evade immune surveillance. Blocking CTLA-4 with monoclonal antibodies such as ipilimumab or tremelimumab can reverse this inhibition and trigger anti-tumor immune responses. These agents have demonstrated safety and efficacy in other cancers and are under investigation for OSCC, especially in combination with other immunotherapies [140,141,142,143].

The PD-1/PD-L1 pathway is another critical target. PD-1 is expressed on activated T cells, interacts with its ligands PD-L1 and PD-L2, and is often upregulated in tumors, including OSCC [144]. Engagement of PD-1 suppresses T cell activation and cytokine production, leading to immune evasion [144,145,146,147]. ICIs such as pembrolizumab and nivolumab (anti-PD-1 antibodies), and atezolizumab and durvalumab (anti-PD-L1 antibodies), have shown promising response rates and improved survival in OSCC, particularly in recurrent or metastatic disease. Pembrolizumab and nivolumab are FDA-approved for recurrent/metastatic HNSCC and are being evaluated in ongoing phase III trials specifically for OSCC.

Several promising immunotherapy clinical trials are currently underway to advance treatment for OSCC and related head and neck cancers. The TACTI-003 trial (NCT04811027) is a Phase IIb study evaluating the combination of eftilagimod alpha (efti), a soluble LAG-3 protein, with pembrolizumab as a first-line treatment for recurrent or metastatic HNSCC, including oral cancers. This trial aims to improve response rates and survival outcomes across PD-L1-positive and -negative subgroups. Another innovative approach is being tested in the TG4050 trial (NCT03839524), a Phase I/II study investigating a personalized neoantigen mRNA vaccine designed to prevent cancer recurrence post-surgery by activating a patient-specific immune response. In parallel, the PRGN-2009 trial (NCT04432597) is assessing a therapeutic HPV vaccine, alone or in combination with M7824 (a bifunctional fusion protein targeting both PD-L1 and TGF-β), in HPV-positive head and neck cancers. Additionally, the ONC-392 trial (NCT04140526) explores an anti-CTLA-4 antibody alone or combined with pembrolizumab in patients with advanced solid tumors, including OSCC, aiming to enhance anti-tumor immunity with reduced toxicity. The NBTXR3 trial (NCT03589339) investigates the use of a nanoparticle radioenhancer activated by radiotherapy, combined with anti-PD-1 therapy (pembrolizumab/nivolumab), to improve immune activation and local tumor control in advanced cancers, including oral cancer. These trials reflect a growing focus on personalized and combinatory strategies to overcome resistance and improve outcomes in oral cancer treatment (Table 3).

Despite these advances, a considerable proportion of patients exhibit either primary or acquired resistance to immunotherapy. Mechanisms underlying resistance are multifactorial and include both tumor-intrinsic and tumor-extrinsic factors [148]. Tumor-intrinsic mechanisms involve loss of antigen presentation (HLA class I loss via immunoediting), JAK/IFNGR1 mutations impairing interferon signaling, and upregulation of LAG3/TIM-3/CTLA-4 checkpoints [149,150]. The tumor-extrinsic factors include the presence of immunosuppressive Tregs, MDSCs, and M2-TAMs in the TME [151]. Metabolic reprogramming (e.g., hypoxia-driven glycolysis) and hypoxia further suppress anti-tumor immunity by polarizing macrophages to M2 and impairing NK/T cell function [152]. These challenges underscore the need for biomarkers (e.g., HPV status, PD-L1 expression) and combination therapies, such as anti-PD1 + STING agonists or hypoxia-targeting agents, to overcome resistance in OSCC [153,154].

Beyond checkpoint inhibition, adoptive cellular immunotherapy offers another innovative strategy, harnessing and enhancing the patient’s own immune cells to target OSCC.

### 4.2. Adoptive Cellular Immunotherapy (ACI)

ACI involves transferring immune cells with enhanced anti-tumor activity into patients [155], either as a standalone treatment or in combination with surgery, radiotherapy, and chemotherapy. Key approaches include Lymphokine-Activated Killer (LAK) cells, Chimeric Antigen Receptor T (CAR-T) cells, CD3 Antibody-Activated Killer (CD3AK) cells, Cytokine-Induced Killer (CIK) cells, and DCs [156].

CAR-T cell therapy, in particular, has shown remarkable efficacy against hematological malignancies by targeting specific tumor antigens [157]. The process involves isolating T cells from the patient’s peripheral blood, genetically modifying them to express CARs that recognize tumor-specific antigens, expanding the modified cells, and reinfusing them into the patient [158] (Figure 4). While CAR-T cell therapy has achieved notable success in blood cancers, its application in OSCC remains in its early stages. Preliminary studies targeting antigens like ErbB and MUC1 offer promising avenues for further exploration in OSCC [159,160].

To enhance the effectiveness of CAR-T cell therapy in OSCC, further research should focus on developing CAR-T cells that target not only tumor-associated antigens (TAA) but also components of the TME that contribute to immunosuppression, such as macrophages, Tregs, CAFs, and IC molecules such as PD-1 and CTLA-4. Overcoming the immunosuppressive TME is essential to improving CAR-T cell efficacy and achieving a durable response in OSCC. In addition to advances in immunotherapy, recent research has highlighted the profound influence of metabolic pathways on immune cell function and tumor progression in OSCC.

## 5. The Role of Metabolism in Shaping Immune Responses

The TME in OSCC is a dynamic metabolic landscape where both tumor and immune cells compete for essential nutrients, metabolites, and signaling molecules. Tumor cells often exploit key metabolic pathways such as glycolysis, the TCA cycle, PPP, and lipid metabolism to sustain their rapid proliferation, suppress immune responses, and evade anti-tumor immunity [161] (Table 4).

For instance, tumor cells upregulate enzymes such as hexokinase 2 (HK2), which not only promotes glycolysis but also increases PD-L1 expression, thereby suppressing CD8^+^ T cell activity and facilitating immune evasion [162,163]. Similarly, lactate dehydrogenase A (LDHA) is frequently overexpressed in OSCC, leading to increased conversion of glucose to lactate. The accumulation of lactate acidifies the TME, impairs NK cell function, and reprograms TAMs toward an immunosuppressive M2 phenotype, ultimately promoting tumor growth and the secretion of pro-tumor cytokines [164,165].

Importantly, the metabolic relationship between tumor and immune cells within the TME is bidirectional. Immune cells actively shape tumor metabolism through nutrient competition and cytokine signaling. For example, TAMs secrete cytokines such as IL-6 and TNF-α, which upregulate glycolytic enzymes like HK2 and LDHA in tumor cells, amplifying lactate production and fostering a pro-tumorigenic environment [163,166]. Conversely, CD8^+^ T cells compete with tumor cells for glucose; their scarcity in the TME can force tumor cells to adapt by increasing glutaminolysis and fatty acid oxidation, further impairing T cell function [167,168]. Tumor-derived metabolites such as succinate can polarize macrophages toward the M2 phenotype, while α-ketoglutarate produced by immune cells enhances T cell immunity by upregulating MHC-I expression, illustrating a complex feedback loop [169,170]. Lipid metabolism also plays a significant role in this interplay; for example, tumor cholesterol sequestration can limit dendritic cell function, and lipid-laden regulatory T cells (Tregs) enforce immune tolerance within the TME [171,172].

The TCA cycle is deeply involved in these metabolic dynamics. Fumaric acid accumulation in CD8^+^ T cells, due to loss of fumarate hydratase, suppresses their anti-tumor capacity [173]. Succinic acid promotes cancer cell migration and invasion by driving macrophage polarization, whereas α-ketoglutarate can enhance T cell function and anti-tumor immunity [169,170]. Glutamine metabolism is also crucial for both tumor and immune cells, influencing T cell proliferation and macrophage activation. Depletion of glutamine or arginine in the TME weakens T cell function, while supplementation can restore anti-tumor immunity by boosting cytotoxic T cell and NK cell activity [167,168,174,175].

Tumor cells further manipulate tryptophan metabolism within the TME. Enzymes like indoleamine 2,3-dioxygenase (IDO) convert tryptophan into immunosuppressive kynurenine, which activates the aryl hydrocarbon receptor (AHR) in CD4^+^ T cells, promoting Treg formation and impairing CD8^+^ T cell function [176,177,178]. This dysregulation of amino acid metabolism contributes significantly to immune evasion and tumor growth.

In addition to carbohydrate and amino acid metabolism, lipid metabolism is pivotal in shaping the TME. Tumor cells often shift toward increased lipid utilization, particularly under glucose or oxygen shortages and cholesterol-rich membranes to evade immune destruction [168]. CD8^+^ T cells in the TME may struggle with long-chain fatty acids, leading to functional exhaustion [171], while high cholesterol levels can enhance NK cell activity [179].

Tregs also rely on lipid metabolism to maintain immune tolerance, and inhibiting their lipid synthesis pathways has been shown to enhance anti-tumor immunity [172]. Collectively, these findings underscore the intricate metabolic interactions within the TME that influence both immune cell function and tumor progression. Tumor cells exploit metabolic pathways to sustain their growth and suppress immunity, but immune cells can, in turn, reshape tumor metabolism through reciprocal signaling and competition. Targeting these bidirectional interactions—such as combining LDHA inhibitors with immune checkpoint blockade or reprogramming TAMs—offers promising strategies for improving immunotherapy outcomes in OSCC. Despite these advances, several challenges remain in translating biomarker discoveries and metabolic insights into reliable clinical tools for OSCC management.

## 6. Comparative Insights with Other Head and Neck Cancers

In contrast to other head and neck squamous cell carcinoma (HNSCC) subsites, OSCC, a common subtype, exhibits both overlapping and unique immunological and metabolic characteristics.

Epidemiologically, OSCC and other head and neck cancers show distinct patterns in incidence and risk factors. OSCC remains one of the most common head and neck malignancies globally (approximately 380,000 new cases annually), exceeding the incidence of oropharyngeal (~98,000 cases), nasopharyngeal (~133,000), and laryngeal (~185,000) cancers [180] Traditional risk factors for OSCC including smoking, chewing betel quid, and using smokeless tobacco differ from the viral etiologies that dominate certain other subsites. Nasopharyngeal carcinoma is mainly linked to Epstein Barr virus and exhibits a distinct geographic prevalence, especially in Southeast Asia and southern China, whereas oropharyngeal squamous cell carcinoma is often linked to infections by high-risk human papillomavirus (HPV) [181,182]. These etiologic differences translate into distinct demographic trends. OSCC and laryngeal SCC typically occur in older individuals with a male predilection (reflecting historically higher exposure to tobacco and alcohol in men) [183]. In contrast, HPV-associated oropharyngeal cancer often presents in relatively younger patients and, according to recent reports, includes more cases in females and non-smokers than traditional HNSCC [183]. Survival outcomes also vary. HPV-positive oropharyngeal carcinomas have a markedly better prognosis than their HPV-negative counterparts when treated with standard therapy. NPC, despite a high propensity for nodal metastasis, is generally chemosensitive and radiosensitive—with concurrent chemoradiation achieving excellent control in non-metastatic disease. By comparison, OSCC tends to exhibit more aggressive locoregional behavior; stage-for-stage oral cavity cancers often have worse disease-specific survival rates than cancers of the larynx or pharynx [184]. Overall, the five-year survival rates for head and neck squamous cancers remains around ~50% on average [185], but this figure belies the considerable prognostic heterogeneity between subsites (e.g., favorable outcomes in HPV-mediated OPSCC versus persistently poorer outcomes in advanced OSCC or hypopharyngeal disease).

Immunologically, OSCC is distinguished by a suppressive TME that is abundant in MDSCs, CD163^+^ M2 macrophages, and increased neutrophil extracellular traps (NETs). These factors are all associated with immune escape and nodal metastasis [186]. While OSCC consistently exhibits abundant and prognostically significant infiltration of CD8^+^ tissue-resident memory T (TRM) cells (marked by CD103, CD69, and CD49a), OPSCCs, particularly HPV-positive cases, exhibit higher levels of tumor-infiltrating CD8^+^ T cells and a better response to immunotherapy [187]. Despite being seen in other locations, these TRM populations have a higher prognostic weight in OSCC, highlighting the possibility of immunotherapeutic targeting through checkpoint pathways such PD-1, CTLA-4, and TIM-3.

Although OSCC and other head and neck squamous malignancies have some common metabolic changes, including elevated fatty acid production, altered amino acid metabolism, and increased glycolysis [188], OSCC exhibits a greater reliance on aerobic glycolysis and elevated lactate export. Additionally, it exhibits clear alterations in choline-containing substances that may be found in saliva, such as propionylcholine and decanoylcarnitine, which could be used as non-invasive biomarkers to differentiate between precancerous and cancerous tumors [39]. OSCC more directly connects the tryptophan to kynurenine pathway to the activation of regulatory T cells and the upregulation of PD-L1, which further contributes to its immunosuppressive environment, even though dysregulation of this pathway aids in immune escape across head and neck cancers [34].

Therefore, unique metabolic reprogramming and an even more restrictive immunological milieu emphasizes its usefulness for targeted therapy and precision diagnosis in OSCC.

## 7. Challenges and Limitation for Biomarkers Research

Research on biomarkers for OSCC faces numerous challenges spanning biological, technical and translational domains. One persistent obstacle is the significant molecular and clinical overlap between OSCC and other oral pathologies, such as Leukoplakia and Oral Lichen Planus. These non-malignant conditions often exhibit similar changes in gene expression, cytokine profiles, and epigenetic modifications, making it difficult to distinguish between benign, pre-malignant, and malignant lesions based solely on biomarker data. For example, inflammatory mediators like IL-6 and IL-8, as well as methylation changes in genes such as SHOX2 and SEPT9, are not exclusive to OSCC and can also be elevated in chronic inflammatory states, reducing the specificity of these markers for cancer detection [76,189,190]. This challenge is compounded by the influence of local inflammation, tissue sampling variability, and prior treatments, all of which can alter biomarker levels and confound the interpretation of results. Another major barrier is the intrinsic heterogeneity of OSCC. Tumors arising in different anatomical subsites of the oral cavity, or even within the same lesion, can harbor distinct genetic mutations, copy number variations, and epigenetic landscapes. This diversity is reflected in the variable expression of oncogenes, tumor suppressors, and non-coding RNAs, such as miR-21 and miR-155, across patient populations and disease stages. As a result, biomarkers that perform well in one subgroup may lack sensitivity or prognostic value in others, limiting their generalizability and clinical utility [191,192]. Such kinds of heterogeneous and contradictory results are common in biomarker research, largely due to population variances, differences in study design, and technological heterogeneity, which further complicate interpretation and clinical translation. Therefore, to enhance evidence-based findings in the field of biomarkers for OSCC, we suggest that more systematic reviews and meta-analyses should be conducted that can rigorously evaluate the study quality and their clinical applicability.

The dynamic nature of tumor evolution, including the emergence of resistant clones during therapy, further complicates the identification of stable and reliable biomarkers. Technical and methodological issues also present significant hurdles. The lack of standardized protocols for sample collection, processing, and analysis often leads to inconsistent findings across studies. Differences in the use of biological matrices (e.g., saliva, plasma, tissue biopsies), isolation techniques (such as those for circulating tumor cells or exosomes), and detection platforms (ranging from ELISA to next-generation sequencing) can all impact the reproducibility and comparability of results. Furthermore, the increasing use of high-throughput, multi-omics technologies generates vast and complex datasets that require sophisticated bioinformatics tools for integration and interpretation. However, many studies are limited by small sample sizes and lack validation in independent or diverse cohorts, increasing the risk of overfitting, publication bias, and false-positive discoveries [68].

Translating biomarker discoveries into clinical practice is further impeded by regulatory, logistical, and economic barriers. Many candidate biomarkers, including promising long non-coding RNAs and protein panels, have yet to undergo rigorous multicenter validation or demonstrate clear clinical benefit in prospective trials. The absence of harmonized guidelines for biomarker qualification and the high costs associated with advanced molecular assays also limit their adoption in routine clinical workflows.

To overcome these formidable challenges, future research must embrace multi-dimensional and collaborative strategies. The development of integrated biomarker panels that combine genomic, epigenetic, transcriptomic, and proteomic data holds promise for capturing the full complexity of OSCC and improving diagnostic and prognostic accuracy. Advances in liquid biopsy technologies, including standardized protocols for ctDNA and exosome analysis, may enhance the sensitivity and reproducibility of non-invasive tests [145,193]. Artificial intelligence (AI) and machine learning approaches are increasingly being leveraged to analyze large-scale, multi-omics datasets, enabling the identification of novel biomarker signatures that are robust across different patient populations and disease contexts. Importantly, rigorous validation in large, ethnically diverse cohorts, the adoption of standardized methodologies, and the establishment of collaborative consortia will be essential to ensure that biomarker discoveries are reproducible, clinically relevant, and applicable to real-world settings [194,195,196].

As the field continues to evolve, integrating multi-omics technologies and AI holds great promise for overcoming current limitations and advancing precision oncology in OSCC.

## 8. Future Directions: Emerging Technologies in OSCC Biomarker Research

The integration of AI-driven biomarker discovery and spatial transcriptomics is revolutionizing OSCC research by addressing tumor heterogeneity and improving biomarker specificity [197]. Deep learning models now analyze multi-omics datasets to identify novel signatures with unprecedented accuracy, including for oral cancer and salivary biomarker discovery [198]. For example, AI frameworks trained on histopathology slides can predict disease presence and classify lesions, enabling non-invasive mapping of the tumor microenvironment and identification of biomarkers linked to invasion or therapy resistance [199]. Spatial transcriptomics resolves intra-tumoral heterogeneity at single-cell resolution, revealing distinct transcriptional programs in the tumor core versus leading edge regions. In OSCC, leading edge-specific gene signatures correlate with poor prognosis and conserved immune evasion mechanisms, while tumor core profiles reflect tissue-specific metabolic adaptations [197]. Platforms such as Path2Space exemplify the synergy of AI and spatial biology, predicting TME cell-type abundances and drug response directly from histopathology images, as demonstrated in breast cancer research [200]. These technologies also enhance liquid biopsy utility: AI models analyzing multi-omics salivary biomarkers offer real-time monitoring of oral cancer progression and treatment response [198].

Future efforts should prioritize validating these tools in diverse patient cohorts, standardizing AI-driven biomarker panels, and leveraging spatial-omics to design therapies targeting critical TME niches. For instance, AI models predicting spatial heterogeneity in breast cancer could be adapted to OSCC to stratify high-risk patients [200]. Similarly, spatial transcriptomics-guided interventions disrupting leading edge-specific pathways may improve survival outcomes. By bridging molecular insights with clinical applicability, these innovations promise to transform OSCC management through earlier detection, personalized treatment, and dynamic therapeutic monitoring [197,198].

## 9. Conclusions 

In the rapidly evolving field of cancer therapeutics, biomarkers have become pivotal tools driving the transition towards precision oncology in OSCC. The integration of immune-profiling and metabolic biomarkers with cancer therapies has enhanced treatment outcomes by enabling more accurate patient selection and the development of tailored therapeutic strategies. Predictive biomarkers such as PD-L1 expression and tumor mutational burden are increasingly used to optimize immunotherapy efficacy, while altered metabolic profiles within tumors offer new avenues for targeted interventions.

Despite significant progress, important gaps remain in our understanding of the TME, particularly regarding the metabolic reprogramming of specific cell populations. This calls for the need to focus on multi-omics approaches and spatial transcriptomics to unravel tumor heterogeneity and the complexity of the TME. Future research must prioritize standardization of multi-marker signatures that combine genomic, proteomic, and metabolomic biomarkers in saliva- and blood-based assays for clinical utility.

## Figures and Tables

**Table 1:** Metabolic Reprogramming in OSCC: Diagnostic Biomarkers and Therapeutic Targets.

Metabolic Pathway/Biomarker	Key Alterations in OSCC	Diagnostic Methods/Biomarkers	Clinical/Diagnostic Performance	Therapeutic Targets/Implications
Amino Acid Metabolism	↓ Aspartate, glutamate, arginine; ↑ asparagine, glutamine, cysteine; tryptophan metabolism via kynurenine pathway enhances Treg function	Plasma free amino acid profiling (HPLC, UHPLC/Q-Orbitrap HRMS); urine metabolite analysis; Mendelian randomization studies	Plasma panel (decanoylcarnitine, cysteine, cholic acid): AUC 0.998; urine markers: sensitivity 94.4%, specificity 91.4%	SLC1A5/ASCT2 (glutamine transporter) inhibition; targeting tryptophan metabolism
Lipid/Choline Metabolism	↑ Phosphatidylcholine, phosphatidylethanolamine; ↓ free choline; ↑ trimethylamine N-oxide; ↑ FA uptake (CD36, FABP5); ↑ FASN	NMR, UHPLC/Q-Orbitrap HRMS, salivary metabolomics	Salivary propionylcholine: sensitivity 76.9%, specificity 96.7%; multi-marker AUC 0.997	FASN, S1PR2, CD36, FABP5, SREBP1, ACC inhibitors
Glycolysis (Warburg Effect)	↑ Glycolysis/lactate production; ↑ HK-II, GLUT-1/3, MCT-4 expression; ↑ serum pyruvic acid	Serum pyruvic acid measurement; enzyme/transporter expression analysis	Serum pyruvic acid: OSCC 2.65 mg% vs. controls 0.95 mg% (*p* < 0.0001)	HK-II inhibition (2-DG), GLUT-1/3, MCT-4 inhibitors
Pentose Phosphate Pathway (PPP)	↑ G6PD expression; ↑ NADPH and ribose-5-phosphate production; supports redox and nucleotide synthesis	G6PD expression analysis	G6PD correlates with advanced disease, poor prognosis	G6PD inhibition (e.g., polydatin); PPP inhibition restores chemosensitivity
Salivary Metabolites	↑ Glutamate, histidine, sialic acid, TMAO, malic acid, methionine, inosine, guanine, spermidine, pipercolate; ↓ urea	CE-MS, CPSI-MS, machine learning panels, salivary metabolomics	Multi-marker panels: up to 100% sensitivity, AUC 0.997; decanoylcarnitine: AUC >0.95, 97.3%	Non-invasive diagnosis, early detection, large-scale screening

↑ for upregulated and ↓ for downregulated.

**Table 3:** List of ICIs approved or under investigation for OSCC.

Clinical Trial Name/Number	Cancer Type	Treatment Regimen	Clinical Phase/Approval	Key Findings/Focus
**KEYNOTE-048** **NCT02358031**	Recurrent/metastatic HNSCC (incl. OSCC)	Pembrolizumab ± chemotherapy	Phase III FDA-approved	Improved overall survival (OS), especially in PD-L1+ patients; fewer severe AEs than chemotherapy.
**NBTXR3** **NCT03589339**	Locally advanced HNSCC (incl. OSCC)	NBTXR3 + radiotherapy + anti-PD-1	Phase I	40% ORR, 75% disease control; effective in anti-PD-1-resistant cases; safe and durable responses.
**TACTI-003** **NCT04811027**	Unresectable recurrent/metastatic HNSCC	Eftilagimod alpha + pembrolizumab	Phase IIb	Dual immunotherapy (LAG-3 + PD-1); evaluating efficacy in PD-L1-low tumors.
**ONC-392** **NCT04140526**	Advanced solid tumors (incl. OSCC)	Anti-CTLA-4 (ONC-392) ± pembrolizumab	Phase I/II	Novel CTLA-4 inhibitor with reduced toxicity; under evaluation for advanced/refractory disease.
**AGEN1181** **NCT03860272**	Advanced solid tumors (incl. OSCC/HNSCC)	Fc-enhanced anti-CTLA-4 (AGEN1181) ± AGEN2034 (PD-1)	Phase I/II	Engineered CTLA-4 therapy aiming for broader immune activation and better safety profile.
**PRGN-2009** **NCT04432597**	HPV-positive HNSCC (incl. OSCC)	Therapeutic HPV vaccine ± M7824	Phase I/II	Boosting HPV-specific T cell immunity; promising for virus-driven oral cancers.
**TG4050** **NCT03839524**	Resected HNSCC (incl. OSCC)	Personalized mRNA neoantigen vaccine	Phase I/II	Individualized vaccine for post-surgical patients to prevent recurrence; early positive immune response.
**Ipilimumab/Tremelimumab** (Various Trials)	HNSCC (incl. OSCC)	Anti-CTLA-4 ± anti-PD-1 (e.g., nivolumab)	Phase I/II	Combination checkpoint blockade; under study for synergy and durable responses.

**Table 4:** Metabolic–Immune Interactions in OSCC: Mechanisms and Therapeutic Opportunities.

Metabolic Pathway	Tumor-Driven Mechanisms	Immune Consequences	Therapeutic Strategies
**Glycolysis**	- Overexpression of HK2/LDHA fuels glycolysis and lactate production. - PD-L1 upregulation via HK2.	- Lactate acidifies TME, impairing NK cells and polarizing TAMs to M2. - PD-L1 suppresses CD8^+^ T cells.	- LDHA inhibitors (oxamate). - Anti-PD-1/PD-L1 antibodies.
**TCA Cycle**	- FH loss → fumarate accumulation (inhibits CD8^+^ T cells). - Succinate promotes M2 polarization.	- α-Ketoglutarate enhances DC/T cell immunity via MHC-I. - Succinate drives immunosuppressive macrophages.	- Restore FH activity. - Modulate succinate/α-KG balance (e.g., α-KG supplementation).
**Amino Acid Metabolism**	- IDO-mediated tryptophan → kynurenine. - Glutamine/arginine depletion in TME.	- Kynurenine activates Tregs via AhR. - Depleted amino acids impair cytotoxic T/NK cells.	- IDO inhibitors (epacadostat). - Glutamine/arginine supplementation.
**Lipid Metabolism**	- Increased FASN-driven lipogenesis. - Cholesterol sequestration in tumor membranes.	- Lipid-dependent Tregs enforce tolerance. - Cholesterol impairs DC antigen presentation.	- FASN inhibitors (orlistat). - Statins to boost NK cell activity.
**Bidirectional Interactions**	- TAM-secreted IL-6/TNF-α upregulates tumor HK2/LDHA. - Tumor succinate polarizes macrophages.	- CD8^+^ T cells compete for glucose, forcing tumor glutaminolysis. - Immune-derived α-KG enhances anti-tumor immunity.	- Reprogram TAMs (lactate modulation). - Combine glycolysis inhibitors + immunotherapy.

## Data Availability

This article does not involve data sharing, as no new data were generated or analyzed in this study.
